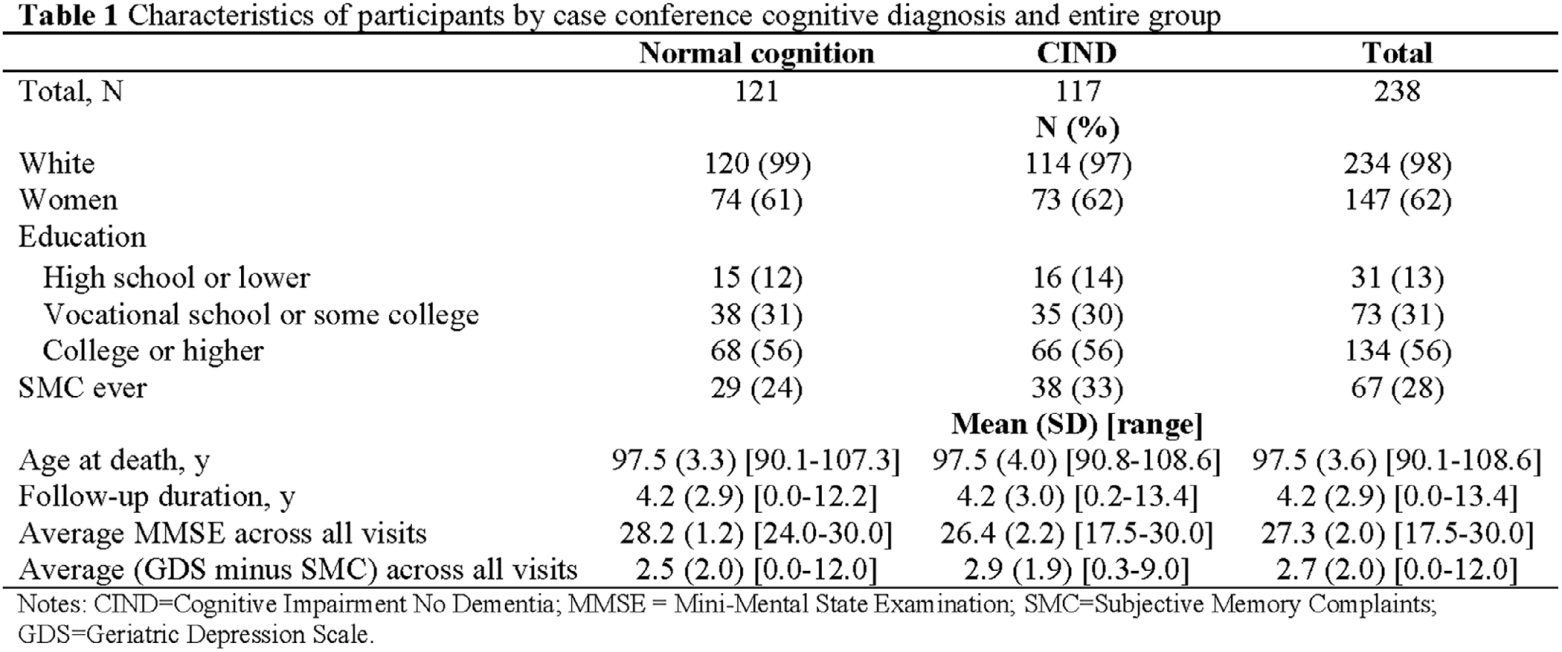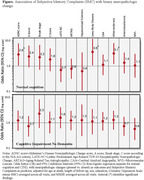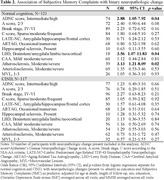# Subjective memory complaints and neuropathologic changes at age 90 and older: *The 90+ Study*

**DOI:** 10.1002/alz.089868

**Published:** 2025-01-03

**Authors:** Zarui A. Melikyan, Claudia H. Kawas, Annlia Paganini‐Hill, Zeinah Al‐Darsani, Luohua Jiang, Syed A. Bukhari, Thomas J. Montine, María M. M. Corrada

**Affiliations:** ^1^ University of California Irvine, Irvine, CA USA; ^2^ University of California, Irvine, Irvine, CA USA; ^3^ UCI, Irvine, CA USA; ^4^ Stanford University, Stanford, CA USA; ^5^ Department of Pathology, Stanford University School of Medicine, Stanford, CA USA

## Abstract

**Background:**

Subjective Memory Complaints (SMC) are defined as the perception of one’s own memory. In several studies SMC are associated with Alzheimer’s disease (AD) neuropathologic changes, and only one study has analyzed and found an association of SMC with other neurodegenerative, but not vascular, neuropathologic changes. Yet, the evidence on the association of SMC with non‐AD neuropathologic changes is insufficient. We aim to examine the association of SMC with neurodegenerative and vascular neuropathologic changes in individuals age 90 and older (oldest‐old) without dementia at death.

**Method:**

The participants of ***The 90+ Study***, a longitudinal study of aging and cognition, who had neuropathologic examination, SMC evaluation, and had no dementia at consensus case conference were included in the analysis. SMC was evaluated with a question from the Geriatric Depression Questionnaire (GDS) “Do you feel you have more problems with memory than most?”, consensus case conference diagnosis was normal cognition or Cognitive Impairment No Dementia (CIND). SMC was dichotomized as reported ever vs. never during the study follow‐up, and neuropathologic changes were dichotomized as present (see Table 2) vs. absent. We estimated odds ratio [OR] and 95% confidence interval [CI] for presence of each neuropathologic change in those with normal cognition and CIND using logistic regression adjusted for demographics, GDS minus the SMC item averaged across all visits, and MMSE averaged across all visits.

**Result:**

In 238 participants mean age at autopsy was 98 years, 121 (51%) had normal cognition, 67 (28%) have ever reported SMC (Table 1). Those with normal cognition and SMC had higher odds of AD neuropathologic change (OR = 2.9; CI: 1.5‐7.9; *p* = 0.04), Lewy Body Disease (LBD) (OR = 3.6; CI: 1.8‐11.8; *p* = 0.04), and atherosclerosis (OR = 3.1; CI: 1.2‐8.1; *p* = 0.02). There were no significant findings in those with CIND (Figure, Table 2).

**Conclusion:**

In the oldest‐old with normal cognition SMC are associated with higher odds of neurodegenerative (AD and LBD) and vascular (atherosclerosis) neuropathologic changes. It is possible that those with normal cognition complain more selectively with respect to neuropathologic changes, compared to those with CIND.